# 1829. Dalbavancin versus standard of care as directed therapy for *Staphylococcus aureus* bacteremia in patients unable to receive OPAT

**DOI:** 10.1093/ofid/ofac492.1459

**Published:** 2022-12-15

**Authors:** J Frazier, Laura Stoudenmire, Jamie Wagner, Geren Thomas, Andrés F Henao-Martínez, Carlos Franco-Paredes, Daniel B Chastain

**Affiliations:** Phoebe Putney Memorial Hospital, Moultrie, Georgia; Phoebe Putney Memorial Hospital, Moultrie, Georgia; University of Mississippi School of Pharmacy, Jackson, Mississippi; John D. Archbold Memorial Hospital, Thomasville, Georgia; University of Colorado Anschutz Medical Campus, Aurora, Colorado; University of Colorado Anschutz Medical Campus, Aurora, Colorado; University of Georgia College of Pharmacy, Albany, Georgia

## Abstract

**Background:**

The purpose of this study was to compare dalbavancin to standard of care (SOC) for patients with *S. aureus* bacteremia (SAB) who were unable to receive outpatient parenteral antimicrobial therapy (OPAT) and would otherwise remain hospitalized or require placement into post-acute care facilities.

**Methods:**

This retrospective cohort compared readmission rates related to the index infection between patients treated with dalbavancin or SOC for SAB between January 1, 2016, and August 31, 2021. Patients at least 18 years old seen by the ID consult service who received at least one dose of dalbavancin or at least one week of SOC parenteral antibacterials as directed therapy for SAB at the time of discharge were included. The SOC group consisted of patients transferred from the main hospital to one of the post-acute care facilities in a surrounding county to complete parenteral antibacterials. Patients were excluded if they were treated for polymicrobial infections with organisms other than *S*. *aureus*, had a creatinine clearance less than 30 mL/min, or required renal replacement therapy. The primary outcome was readmission rates within 30 days after completion of therapy. Secondary outcomes included readmission rates within 90 days after completion of therapy as well as antibacterial regimen adherence defined as achieving goal duration of therapy deemed appropriate by the ID consult service.

**Results:**

During the study period, 27 patients received dalbavancin, and 27 patients received SOC. Baseline demographics were comparable between groups, though more patients in the SOC group had indwelling prostheses or hardware (4% vs 22%). The majority of SAB was caused by MSSA in both groups (56% vs 59%). The most common source was osteoarticular (15% vs 30%) and source control was attempted in a higher percentage of the SOC group who had an identified source (53% vs 86%). Readmission rates in the dalbavancin group were similar to those in the SOC group within 30 days (15% vs 22%, p=0.484) and 90 days (19% vs 22%, p=0.735) after completion of therapy. However, adherence was significantly higher among patients treated with dalbavancin (85% vs 44%, p < 0.05).

**Conclusion:**

Dalbavancin offers similar clinical outcomes to SOC for patients with SAB who are unable to receive OPAT.

**Disclosures:**

**All Authors**: No reported disclosures.

